# Smoking cessation using preference-based tools: a mixed method pilot study of a novel intervention among smokers with low socioeconomic position

**DOI:** 10.1186/s13722-021-00254-6

**Published:** 2021-06-30

**Authors:** Mégane Héron, Anne-Laurence Le Faou, Gladys Ibanez, Brigitte Métadieu, Maria Melchior, Fabienne El-Khoury Lesueur

**Affiliations:** 1 Social Epidemiology Departement, Sorbonne Université, INSERM UMR_S 1136, Institut Pierre Louis d’Épidémiologie et de Santé Publique IPLESP, 27 rue Chaligny, Paris, 75012 France; 2 Centre Ambulatoire d’Addictologie, DMU Psychiatrie et Addictologie, Hôpital Européen Georges Pompidou, APHP.Centre, Paris, France; 3 Fédération Hospitalo-Universitaire Network of Research in Substance Use Disorder, Université de Paris, Paris, France; 4 Department of General Practice, Sorbonne Université, Paris, France; 5 Association Charonne, Paris, France; 6 Epidemiology Department, University Hospital Group of Psychiatry and Neurosciences (GHU-Paris), Paris, France

**Keywords:** Smoking cessation, Pilot study, Health disparities, NRT, Electronic cigarette

## Abstract

**Background:**

Compared to smokers with favorable socio-economic position (SEP), those with low SEP are less likely to have a successful smoking cessation attempt. Tailored approaches are therefore needed, and general practitioners could help reaching and assisting usually hard-to-reach population.

**Method:**

STOP (Sevrage Tabagique à l’aide d’Outils dédiés selon la Préférence) is a pilot study, examining the feasibility, acceptability and potentiality of a smoking cessation intervention centered on smoker’s preference. Smokers with low SEP, wishing to quit, were recruited in six healthcare centers in the Greater Paris area. They were asked to choose between different types of nicotine replacement therapy (NRT) products and/or e-cigarette with liquids delivered free of charge to aid their smoking cessation attempt. We describe the characteristics of recruited participants, their perception of smoking cessation aids, and the evolution of their smoking status 4 to 6 weeks after recruitment.

**Results:**

We recruited 49 participants, of which 29% chose an e-cigarette, 29% chose NRT and 42% chose both an e-cigarette and NRT. The intervention was shown to be acceptable by participants and health professionals. Among the 24 participants followed for at least one month, 14 (28% of all participants) stopped smoking, and 9 (18%) considerably reduced their consumption.

**Conclusion:**

The STOP intervention is feasible and acceptable, even if more efforts should be made to limit lost-to-follow-up. This preference-based intervention also shows interesting prospect in helping smokers with low SEP quit smoking. We will test the efficacy of this preference-based intervention in a randomized controlled trial.

## Background

Smoking prevalence among individuals with low socio-economic position (SEP) has remained persistently higher than among persons with high SEP, even with substantial decreases in overall smoking rates in Western countries [1, 2]. This social gradient in smoking is well evident in France, where unemployed adults are twice as likely than employed adults to smoke (39.9% vs. 19.5%) [3]. It is partly explained by lower rates of successful smoking cessation attempts among socially disadvantaged populations [4]. In fact, even if smokers with low SEP are interested in quitting and attempt to quit at rates similar to those of other smokers, they are less likely to succeed [5]. Therefore, “targeted” interventions, designed and aimed at smokers with low SEP are needed, in order to reduce health inequalities. In fact, there is a growing body of evidence suggesting that universal interventions (i.e., “non-targeted” intervention strategies aimed at the entire population) may widen social health inequalities [6].

Moreover, smokers with low SEP tend to have higher levels of nicotine dependency than other smokers [5], and therefore might benefit from nicotine replacement therapy (NRT) to quit or reduce their smoking levels while reducing the discomfort of nicotine withdrawal symptoms. Further, electronic cigarettes (e-cigarettes), have also been shown to aide smoking attempts [7, 8], even if little data on its efficacy among smokers with low SEP is available. In France, even if some NRT products are currently partially refunded by the French national health insurance system [9], smokers with no top-up covering insurance still have to pay at least 35% of the price. Further, the most recent data from the French Health Barometer indicate that smokers with high SEP are more likely to quit and/or use NRT and e-cigarettes to quit smoking than less socially-advantaged smokers [10], Smokers with low SEP, have usually less access to medical information, and might be discouraged from using cessation aids if they have to pay for them (even partly) [11]. Paying upfront for an e-cigarette kit (around 50–80€), or out-of-pocket for part of the NRT price could hinder smoking cessation attempts among smokers with financial difficulties.

Another factor that might contribute to social inequalities in tobacco consumption is health professionals’ implicit bias concerning smokers with low SEP. Studies have shown that health professionals perceive low SEP as a barrier to smoking cessation, [12] and tend to offer less smoking cessation or reduction advice to their most socially disadvantaged patients [13]. Data on health professionals’ perceptions of NRT and e-cigarette are also scarce. A better understanding of their perceptions concerning these tools could help improving smoking cessation interventions in primary care settings.

*Shared decision-making* medicine—where patients are more involved in the medical decision-making process—has been quickly gaining ground among patients and clinicians [14]. Given the existence of different tools for smoking cessation, the personalization of treatments based on smokers’ preference may be more effective than standardized smoking cessation procedures.

This is why we developed “STOP” (*Sevrage Tabagique à l’aide d’Outils dédiés selon la Préférence*: Smoking Cessation using preference-based tools), a smoking cessation intervention aimed at smokers with low SEP. This intervention is led by health professionals and is based on participants’ choice of their preferred smoking cessation aid(s).

In this pilot study, we study the feasibility and acceptability of the STOP intervention in six different centers located in the Paris Area in France, and examine whether preliminary results on smoking cessation are sufficiently encouraging to warrant a randomized controlled trial.

## Methods

### Participants and recruitment

Recruitment took place between June 2018 and July 2019. To be included, individuals had to be ≥ 18 years, smoke at least one cigarette a day, and wish to quit or reduce their tobacco consumption. Participants also had to be unemployed (self-reported) and/or eligible for at least one social benefit reserved to low-income individuals in France. These benefits include the universal health coverage (mandatory health insurance with/without complementary health insurance), medical insurance for undocumented immigrants (AME), the disabled adult allowance (AAH), the minimum social benefits (minimal income, minimal benefit after the unemployed benefits period), the family support allowance (ASF), the family supplement (CF), the housing assistance (APL), and the disability pension.

Exclusion criteria included physical or mental inability to participate in the study as assessed by the health professional, inability to sign a written informed consent in French, inability to communicate in French, or having terminal or life-threatening illness. Pregnant women and smokers undertaking another active smoking cessation therapy at the time of inclusion (pharmacotherapy including NRT or active involvement in a smoking cessation program) were also excluded. Current use (but not past use) of e-cigarettes was also an exclusion criteria.

### Study centers and recruitment

**Fig. 1:**
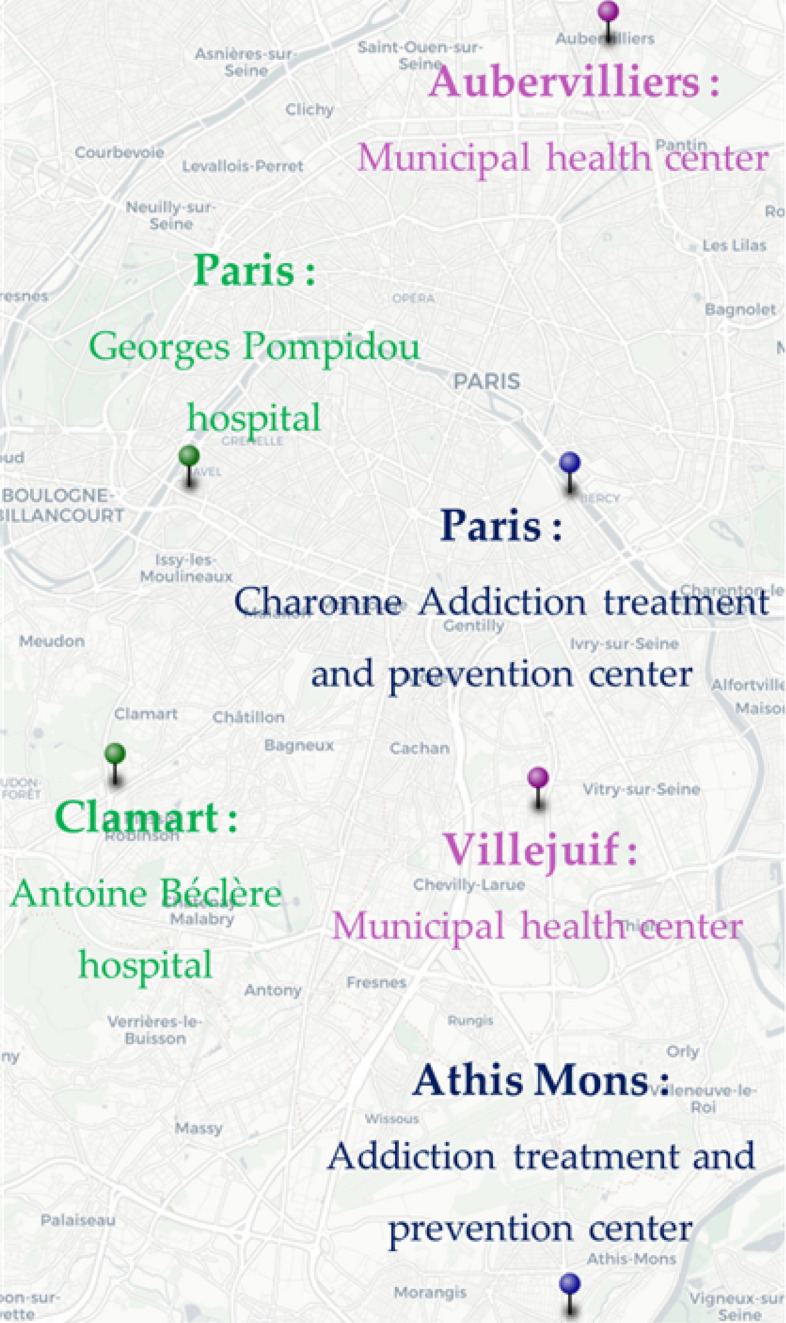
: Participating centers in the STOP pilot study. 2018–2019

The study took place in six centers in the Greater Paris area: two hospitals, two municipal health centers, and two addiction treatment and prevention centers (Fig. 1). In France, people with low SEP are more likely to visit community and municipal health clinics or public hospitals compared to private clinics due to low or lack of out of pocket payments.

Participants were recruited by general practitioners or addiction specialists. After a clear description of the study protocol, eligible individuals who agreed to participate in our study were asked to sign a written informed consent in order to participate.

### Ethical approval

The pilot study obtained the approval from a French medical research ethics committee (Committee of Persons Protections “Comités de Protection des Personnes”; N° CPP 2018–19-1).

### The STOP intervention

The intervention started with a motivational interview, where clinicians asked participants about their smoking status and history of quit attempts, but also about potential obstacles and facilitators for smoking cessation. Then clinician briefly but clearly described existing smoking cessation aids. Available NRT products were: transdermal patch, gum, spray, inhaler, sublingual and tablets/lozenges. Health professionals could also provide an e-cigarette (EnduraT20) along with e-liquids in different flavors (fruits, menthol and tobacco) and nicotine dosages (6 mg/ml, 12 mg/ml and 16 mg/ml). Clinicians adapted the nicotine dosages according to the number of cigarettes smoked by the participant and their smoking history. Participants were therefore asked to choose one or several of those tools to aid their quit or smoking reduction attempt. Products were given in sufficient quantity to last until the following appointment. General smoking cessation advice was given, and expected side effects were also discussed.

Participants were allowed to keep the e-cigarette at the end of the study.

### Baseline assessment and follow-up

Patients were followed for 4 to 6 weeks, with three different appointments: at inclusion, 7 to 10 days, and then 4 to 6 weeks after inclusion, completing a questionnaire at each appointment. At baseline, participants were asked about the number of cigarettes and roll-your-own cigarettes they smoke per day, as well as on their smoking and quit attempt history, and their use of other psychoactive drugs.

Further, data on smoking perceptions of smoking cessation tools (e-cigarettes and NRT), use of psychoactive substances, and socio-demographic characteristics were also self-declared.

We translated and adapted the *Modified Cigarette Evaluation Questionnaire* in order to measure participants’ perceptions of NRT products and e-cigarettes [15]. Participants were therefore asked to answer with either “agree strongly” (1), “agree slightly”(2), “neutral”(3), “disagree slightly”(4), and “disagree very much”(5) to the following questions: “Do you think that NRT or e-cigarette …” a) help to not smoke, b) is as good as the cigarette for health, c) is acceptable for smokers, d) has a more positive social perception than smoking; e) is effective in smoking cessation. The summed scores were later classified into three categories: positive attitude (< 9), neutral/undecided (9–13 for e-cigarette and 9–12 for NS) and negative (≥ 14 for e-cigarette and ≥ 13 for NRT). In case of missing data on more than one item, the score was classified as missing (n = 11 for NRT and n = 8 for e-cigarette). Five participants had missing data on one question, and were classified according to the responses on the other four responses.

Further, anxiety and depressive symptoms were measured with the Hospital Anxiety and Depression Scale (HADS) [16]. The HADs scale consists of two sub-scales (anxiety and depression) with 7 statements each and 4 possible responses ranging from 0 to 3 and all relevant items were summed, and the recommended cut-off points were used to create three separate categories of symptoms of anxiety or depression: absence (< 7), possible cases [8–10], and definite cases (≥ 11) [17].

Anxiety and depressive symptoms are linked to smoking and quit attempts, [18] and information on these symptoms could also be useful for adapting clinicians’ customized smoking cessation advice.

Finally, we measured the perception of having professional difficulties among participants who are employed.

At each follow-up, participants self-reported their smoking status (cessation, reduction, maintenance) as well as their other substance use.

### Health professionals’ questionnaire

Prior to the initiation of study recruitment procedures, health professionals working in participants centers and able to prescribe NRT, filled a short questionnaire describing their opinions regarding smoking cessation tools. We also asked them how often and in which situations, in their regular practice, they talk about NRT and e-cigarettes as smoking cessation aids with their patients who smoke. Likert-type scale was used to measure health professionals’ perceptions regarding e-cigarettes; this score was classified into three categories: positive attitude (< 11), neutral/undecided [11–15] and negative (> 15).

### Qualitative research

One-to-one qualitative research interviews were also conducted with volunteer patients and health professionals, a couple of months after the completion of the recruitment phase. The full analysis of these interviews will be the subject of a future article.

We report two short extracts of transcripts from two interviews, which serve as examples: an interview with a participant who successfully quit smoking, and a second one with a clinician.

### Analyses

We first carried out a descriptive analysis of patients’ characteristics overall and according to patients’ follow-up status (lost to-follow-up Y/N). We described continuous variables by their mean and standard deviation, while categorical data were reported as frequencies (percentages).

## Results

We recruited 49 patients, mostly men (61%), with an average age of 46 (sd = 11.5), and more than half of whom were unemployed. At the time of inclusion, participants who smoked manufactured cigarettes (n = 35) reported smoking an average of 19 (sd = 10) cigarettes per day, while smokers of roll-your-own tobacco (n = 14) smoked an average of 16 cigarettes (sd = 10) per day (Table 1).

**Table 1:** Characteristics of participants of the STOP pilot study

	Total population N = 49
Center	
Athis Mons (addiction treatment and prevention center)	3
Aubervilliers (municipal health center)	6
Charonne (addiction treatment and prevention center)	12
Clamart (hospital)	4
Georges Pompidou (hospital)	13
Villejuif (municipal health center)	11
Sex	
Female	19 (38.8%)
Male	30 (61.2%)
Educational level	
< High school degree	29 (63.0%)
> = High school degree	18 (37.0%)
Missing	3
Professional difficulties	
Yes	9 (19.6%)
No	8 (17.4%)
Did not work at inclusion	29 (63.0%)
Missing	3
Symptoms of depression (HAD)	
Absence	6 (13.3%)
Possible case	14 (31.1%)
Definite case	25 (55.6%)
Missing	4
Symptoms of anxiety (HAD)	
Absence	16 (35.6%)
Possible case	19 (42.2%)
Definite case	10 (22.2%)
Missing	4
Has felt the need to reduce alcohol consumption	
Yes	17 (42.5%)
No	23 (57.5%)
Missing	9
Current use of cannabis	
Yes	14 (30.4%)
No	32 (69.6%)
Missing	3
Current use of another drug	
Yes	8 (18.6%)
No	35 (81.4%)
Missing	7

N = 49, 2018–2019

Characteristics of participants according to their follow-up status are presented in Appendix 1.

### Perception of smoking cessation tools

One-fifth (21%) of STOP pilot study participants had a positive perception of NRT, 51% had a neutral/ undecided opinion, and 28% had a negative perception of these products.

Similar patterns were found for the perception of e-cigarettes, 26% of participants had a positive perception, 48% were neutral/ undecided, and 28% of participants had a negative perception of this tool.

### Choice of smoking cessation tool(s)

Around one in four participants chose an e-cigarette (n = 14, 29%) and NRT (n = 14, 29%) to aid their smoking cessation or reduction attempt, and 42% (n = 20) chose both types of smoking cessation tools. One participant did not report his preference.

Most participants (73%) who had a negative perception of e-cigarettes chose NRT. Participants who reported a neutral/undecided view of e-cigarettes mostly chose it as a smoking cessation or reduction aid. Most participants with a neutral/undecided perception regarding NRT also ended up choosing it (40% alone and 35% along with e-cigarette) (Appendix 2)

### Cigarettes use during follow-up

**Fig. 2:**
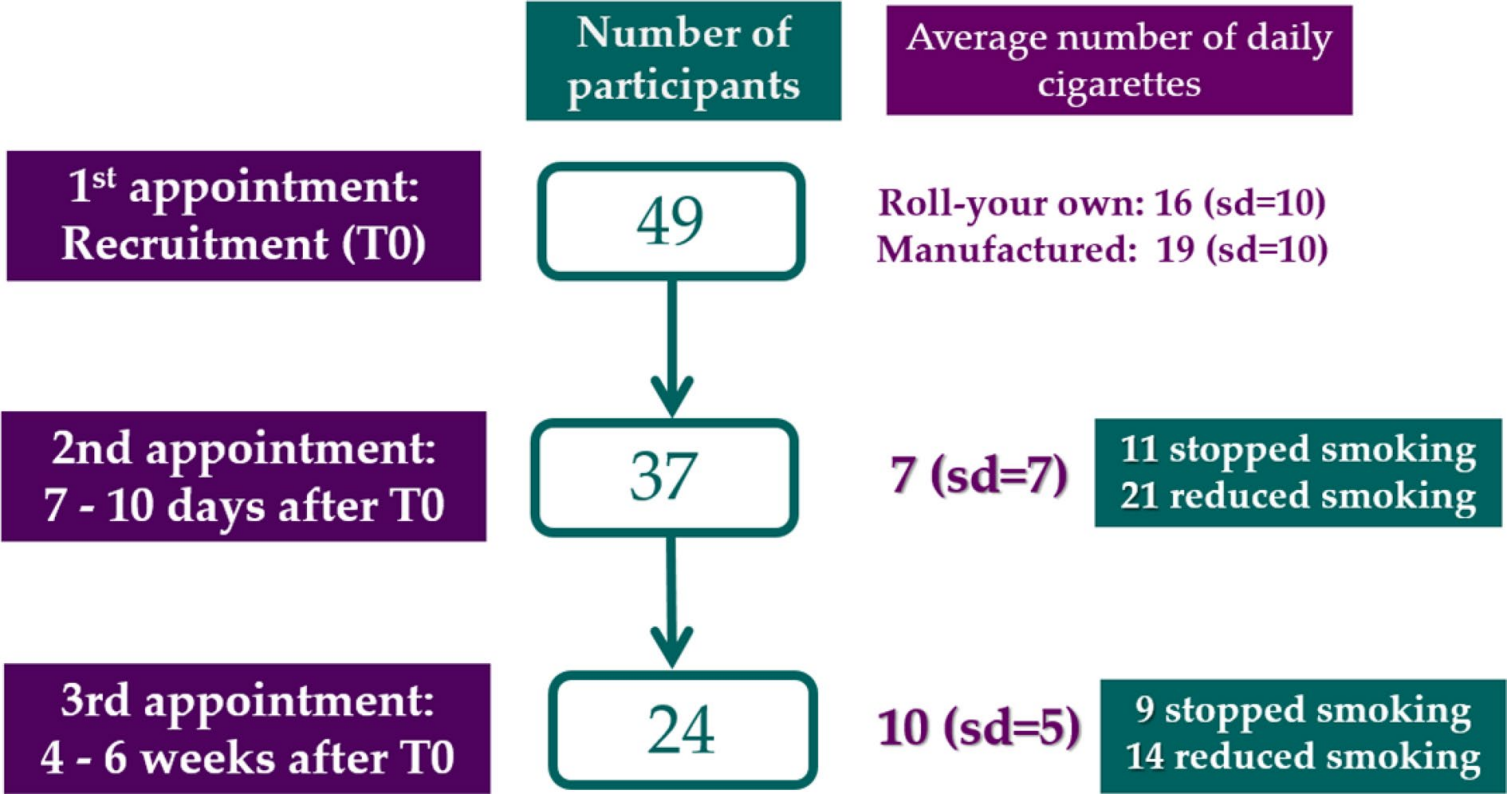
Flow chart of the STOP Pilot study

Follow-up data at the second appointment for 37 participants shows that 11 (30%) participants achieved complete smoking cessation and 21 (57%) significantly reduced their tobacco consumption. The average number of cigarettes smoked significantly decreased from 19.2 (sd = 10) at inclusion to 7.7 (sd = 7) at the second appointment. Five participants (13%) reported having an unchanged cigarette consumption (Fig. 2).

Follow-up data collected at the third appointment for 24 participants show that 14 (58%) participants achieved complete smoking cessation and 9 participants (38%) significantly reduced their tobacco consumption. One participant did not report any reduction in his tobacco consumption. The average number of cigarettes smoked increased between the 2nd and the 3rd follow-up appointment: 10.3 (sd = 5).

In one of the qualitative interviews we conducted, a female former smoker reported: “I participated in this study only in order to get a free e-cigarette. Before the study, I smoked 10 roll-your-own cigarettes per day. I tried several times to quit smoking using NRT products. I tested everything but nothing worked for me. I have never been able to quit smoking despite my attempts. Without this study, I could never have tried the e-cigarette because I don’t have the budget to buy one. I have stopped smoking for 3 or 4 months now. I currently use the e-cigarette daily. I only have one fear right now, that it will break because I would not have the means to buy another one. Therefore I am very careful with it. I find that the e-cigarette is a good replacement for cigarettes. I’m glad I could regain my taste and smell senses, and I feel much better physically. “

### Health professionals’ questionnaire

Seventeen health professionals (three general practitioners, eleven addiction specialists and three other professionals—psychologist, psychiatrist, and a nurse) completed the study questionnaire designed for health professionals.

A majority (59%) of health professionals reported routinely talking about NRT for smoking cessation to their patients who smoke. When patients want to stop smoking, 31% of health professionals usually prescribe NRT, 44% prescribe NRT often, 13% sometimes and 12% rarely or never.

One in 4 health professionals reported mentioning e-cigarettes when they talk about smoking cessation with their patients who smoke. When patients wish to quit, 19% of health professional always recommend using an e-cigarette, 38% recommend it very often, 19% sometimes, and 25% only rarely.

Health professionals also reported their views concerning different advantages and barriers of e-cigarettes and NRT (Table 2).

**Table 2:** Clinicians’ perceptions concerning NRT and e-cigarettes in the context of smoking cessation

	Price	False beliefs participants might have	Complicated to use	Misuse/incorrect application	Different effect than a cigarette	Does not suit everyone	No knowledge about long term effects
NRT	Yes 100% N = 16	No 37.5% Yes 62.5% N = 16	No 81.3% Yes 18.7% N = 16	No 62.5% Yes 37.5% N = 16	No 31.3% Yes 68.7% N = 16	No 87.5% Yes 12.5% N = 16	
E-cigarette	No 35.3% Yes 64.7% N = 17	No 41.2% Yes 58.8% N = 17	No 58.8% Yes 41.2% N = 17	No 82.3% Yes 17.7% N = 17	No 47.1% Yes 52.9% N = 17	No 58.8% Yes 41.2% N = 17	No 77.7% Yes 22.2% N = 9

STOP pilot study N = 17

One fifth of health professionals (n = 2) had a positive perception of e-cigarettes. More than 60% (n = 10) of health professionals had a neutral perception/ were undecided, while 25% (n = 4) had a negative perception. Clinicians’ opinions on different aspects of e-cigarette are reported in Appendix 3.

A female health professional we interviewed reported the following: “I work with persons with low SEP and I observe that they are often too ill-informed to be able to successfully quit smoking. I talk to them all the time about different [smoking cessation] tools and I adapt my suggestions according to their preferences. Most of my patients have other addictions, and tobacco is often forgotten and untreated. I strongly recommend NRT to my patients because scientific data has proven its effectiveness. With this study, I also offer them an e-cigarette if they wish. However, these are patients who smoke a lot, so I always add NRT in addition to the e-cigarette when I can provide it. Therefore, I find it important to have a supply of NRT on hand to give it out. People with a low SEP can successfully quit smoking if they are well surrounded and informed. In this intervention, the possibility of having free nicotine sprays and inhalers was very appreciated because these products are not yet reimbursed.“

## Discussion

The objective of the STOP pilot study was to evaluate the feasibility of a smoking cessation intervention based on persons’ preferred treatment and free dispensation among smokers with low SEP. We also endeavored to examine whether preliminary smoking cessation results are sufficiently encouraging to warrant a randomized controlled trial.

The results show that our intervention is feasible and acceptable in different healthcare settings, and shows promising effects on smoking cessation rates in this population with high levels of smoking. At one month follow-up, around one third (28%) of participants who were included had stopped smoking. These results are very encouraging because not all recruited participants intended to stop smoking at the start of the study.

However, our pilot study highlighted the challenge of this intervention for health professionals, most of whom participated in a research study for the first time, and had difficultly following participants through several appointments spread over weeks. Thus, the percentage of participants lost to follow-up was significant, which could be a potential source of bias in a future larger study since smokers who are struggling or failing in their smoking cessation attempt might be more likely to be lost to follow-up. Nevertheless, even under the assumption that participants who were lost to follow-up did not succeed in quitting smoking, our results are still very promising. In fact, 7 to 10 days after study inclusion (2^nd^ appointment), just under half (42%) of recruited participants reported a reduction in their cigarette consumption, and 22% had stopped smoking. At the one-month follow-up, 18% reduced their cigarette consumption, and 28% stopped smoking.

Physical symptoms of nicotine withdrawal are more important in the first month after nicotine cessation. Therefore, patients who have achieved smoking cessation during the study period should be less likely to relapse to smoking due to withdrawal symptoms. However, the follow-up time of the study is short and does not guarantee long term successful smoking cessation.

The high rate of loss-to-follow-up was a major result of this pilot study, which questions the feasibility of future studies. As a result, we introduced several measures to promote retention in the randomized controlled trial testing the efficacy of the intervention evaluated in this pilot study. These measures include having the possibility to call participants who miss one of the follow-up visits, and giving participants pre-stamped “calendar cards”, on which they can fill in their daily tobacco use, and then send them to the research team.Another novel aspect of our pilot study is the analysis of health professionals’ perceptions concerning different tools available for smoking cessation. They all perceived price as the main barrier for the use of NRT products. In fact, our intervention took place right before many NRT products became partly covered by the French health insurance system. For the majority of health professionals, the other two important barriers were false beliefs about NRT and the fact that they do not provide the same effect as smoking. For the e-cigarette, only price and false beliefs stood out as barriers to their use. This information is very useful for the design of the future STOP trial, regarding the ways in which the intervention should be presented to potential participants, and the information that needs to be supplied concerning smoking aid tools.

Health professionals also highlighted difficulties concerning the length of the questionnaire, which was perceived as “too long”. Thereby, questionnaires were not completed in full and sometimes important information was missing. Moreover, due to the length of the questionnaire, they did not always take the time to include patients for fear of not having enough time to complete them. As a result of these findings, we reduced the length of the questionnaires for the planned randomized controlled trial.

The relatively small number of included participants, and the loss of half of the participants at the 3rd appointment are major limitations. Another limitation is the fact that most participants included in the qualitative study were smokers who quit. In addition, tobacco consumption was self-reported, which could also have resulted in under-reporting.

## Conclusion

Despite difficulties in recruiting a large number of patients and loss to follow-up rate, the results of this pilot are encouraging. Around 1 in 4 low SEP smokers recruited stopped smoking one month after inclusion, and the preference-based intervention was shown to be acceptable to participants and health professionals. This study also provided valuable information that will allow us to optimize the design of a randomized controlled study to test the efficacy of the STOP intervention.

## Data Availability

The datasets used and/or analysed during the current study are available from the corresponding author on reasonable request.

## Appendix 1

See Table 3.

**Table 3:** Characteristics of participants lost to follow-up and those not lost to follow-up

	Patients not lost to follow up n = 24	Patient with 2 follow-up n = 13	Patient with 1 follow-up n = 12
Sexe			
Female	7 (29.2%)	3 (23.1%)	9 (75.0%)
Male	17 (70.8%)	10 (76.9%)	3 (25.0%)
Education level			
Inferior baccalaureate	16 (69.6%)	7 (53.9%)	6 (60.0%)
Superior baccalaureate	7 (30.4%)	6 (46.2%)	4 (40.0%)
Missing	1		2
Professionals difficulties			
Yes	1 (4.4%)	2 (16.7%)	5 (45.5%)
No	7 (30.4%)	1 (8.3%)	1 (9.1%)
Doesn’t work	15 (65.2%)	9 (75.0%)	5 (45.5%)
Missing	1	1	1
Consumption of manufactured cigarettes	17.4 (sd = 9)	14.7 (sd = 8.7)	26.5 (sd = 12.3)
Consumption of roll your own tabacco	15 (sd = 13)	17 (sd = 8.5)	17
Consumption of cannabis			
Yes	6 (25.0%)	7 (53.9%)	1 (11.1%)
No	18 (75.0%)	6 (46.1%)	8 (88.9%)
Missing			3
Current use of another drug			
Yes	6 (27.3%)	2 (16.7%)	0
No	16 (72.7%)	10 (83.3%)	9 (100.0%)
Missing	2	1	3

## Appendix 2

See Table 4.

**Table 4:** Choice of tool based on their perceptions of different tools

Score SN	NRT choice	E-cig choice	Both
Positive perception	3 (37.5%)	2 (25.0%)	3 (37.5%)
Neutral perception	8 (40.0%)	5 (25.0%)	7 (35.0%)
Negative perception	2 (15.4%)	5 (45.6%)	4 (36.4%)

Score e-cig	NRT choice	E-cig choice	Both
Positive perception	2 (18.2%)	4 (36.4%)	5 (45.5%)
Neutral perception	2 (10.0%)	8 (40.0%)	10 (50.0%)
Negative perception	8 (72.7%)	1 (9.1%)	2 (18.2%)

## Appendix 3

See Table 5.

**Table 5:** Health professional’s opinions concerning e-cigarette

	There is a lack of evidence regarding the safety of this product in the long term	Absence of regulatory controls on vaping liquid	It’s potentially a gateway to smoking for teens	It use can perpetuate he dependence of smokers	A very restrictive regulation of this product may limit tobacco harm reduction strategies
Strongly agree	29%	13%	12%	0%	25%
Agree	29%	44%	24%	24%	38%
Neutral / undecided	6%	25%	6%	35%	25%
Disagree	24%	6%	35%	35%	6%
Strongly disagree	12%	12%	23%	6%	6%

## Abbreviations

AAH: ‘*allocation aux adultes handicapés’* (French disabled adult allowance)
AME: ‘*aide médicale de l'État’* (French medical insurance for undocumented immigrants)
APL: ‘*aides personnelles au logement*’ (French disability pension)
ASF: ‘*allocation de soutien familial*’ (French family support allowance)
CF: ‘*complément familial’* (French housing assistance)
CPP: ‘*Committee of Persons Protections’* “Comités de Protection des Personnes’ (French medical research ethics committee).
e-cigarettes: Electronic cigarettes
HADS: Hospital Anxiety and Depression Scale
INCA: ‘*Institut National Du Cancer* ‘ (French cancer institute)
NRT: Nicotine replacement therapy
SEP: Socio-economic position
STOP (study): ‘Sevrage Tabagique à l’aide d’Outils dédiés selon la Préférence’ (Smoking Cessation using preference-based tools
